# Comparison of Body Condition, Milk Production and Animal Movements Between Sopravissana and Comisana Sheep: An Approach to Evaluating Breed Suitability for Sustainable Farming in Central Italy

**DOI:** 10.3390/vetsci13101082

**Published:** 2026-10-13

**Authors:** Sara Moscatelli, Elena De Felice, Martin Wikelski, Federico Maria Tardella, Andrea Catorci, Paola Scocco

**Affiliations:** 1 International School of Advanced Studies, Course “One Health” University of Camerino, Via Madonna Delle Carceri 9, 62032 Camerino, Italy; sara.moscatelli@unicam.it; 2 School of Biosciences and Veterinary Medicine, University of Camerino, Via Pontoni 5, 62032 Camerino, Italy; elena.defelice@unicam.it (E.D.F.); dtfederico.tardella@unicam.it (F.M.T.); andrea.catorci@unicam.it (A.C.); 3 Department of Migration, Max Planck Institute of Animal Behavior, 78315 Radolfzell, Germany; wikelski@ab.mpg.de

**Keywords:** sheep breed, Comisana, Sopravissana, BCS, milk production, movement ecology, sustainable farming

## Abstract

Knowledge of farmed animals’ behavior and productive traits is essential to ensure the economic, environmental, and social sustainability of pastoral activities in a climate change scenario. However, limited information is available on breed differences under semi-extensive farming conditions. This study aimed to assess differences between Comisana and Sopravissana sheep in animal movement, body condition, and milk production. From June to October 2023, 40 female sheep were equipped with GPS-dataloggers to monitor movement patterns during free grazing. During the same season, Body Condition Score (BCS) was repeatedly assessed, while milk production was monitored at the breed-group level in 60 lactating ewes. The two groups showed similar movement patterns during free-grazing times. Milk production differed between the two groups and changed over time, with different temporal trajectories between groups; the Sopravissana group showed lower milk production than the Comisana group but a smaller overall reduction over time. Differences in body condition also emerged, with Sopravissana sheep maintaining a significantly higher BCS than Comisana sheep (*p* < 0.001). Post hoc tests confirmed significant breed differences in both physiological states (lactation/dry) and showed that lactating Sopravissana ewes maintained a higher BCS than dry Comisana ewes (*p* = 0.0002). These findings suggest that the differences observed in body condition and milk-production dynamics may be relevant when considering breed choice in Mediterranean pastoral systems and could contribute to improving sustainable farm management under increasing environmental challenges.

## Simple Summary

Sheep farming in the Mediterranean area is increasingly affected by climate variability. In this context, breed choice may play an important role in maintaining productive and sustainable farming systems. This study compared Comisana sheep, a highly productive breed, and Sopravissana sheep, a traditional local breed from central Italy, to assess differences in movement patterns, body condition, and milk yield. Forty ewes were monitored for movement using GPS collars and BCS during the grazing season, while milk production was recorded at the group level in 60 lactating ewes. Results showed no significant differences between the two breeds in movement patterns during free grazing, while milk-production data showed different patterns in the two monitored groups, with the Sopravissana group producing less milk but showing a smaller overall reduction. Sopravissana ewes also maintained a higher body condition than Comisana ewes throughout the study period. These findings highlight breed-specific differences in body condition and milk production dynamics under the investigated semi-extensive farming conditions. These results suggest that breed-specific characteristics should be considered when selecting animals for Mediterranean pastoral systems.

## 1. Introduction

Mediterranean ecosystems are closely related to human presence after over 10,000 years of “co-habitation,” which makes the conservation of biodiversity inherently linked to human activities [1]. Livestock husbandry and breeding have been of crucial importance for human survival in the Mediterranean area and have become one of the most important forces shaping Mediterranean landscapes [2]. Human activities have been beneficial to the increase in biodiversity in the Mediterranean basin because of the frequent but moderate disturbance applied by domestic herbivores [3,4], which transformed the Mediterranean area into a biodiversity hotspot worldwide [5]. Livestock activity influences the habitat and creates heterogeneous environmental mosaics as a consequence of animals’ trampling, selective grazing, and relocation of seeds. It can also increase facilitation processes of a wide range of plants, particularly those associated with open habitats [6,7].

Sheep (*Ovis aries*) and goats (*Capra aegagrus hircus*) have long been fundamental for human activities in the Mediterranean basin, as they have been domesticated for at least 10,000 years [8]. The region’s diverse topography and climate led to the development of different breeds with distinct characteristics [9]. Over time, sheep breeds were shaped to meet various human needs, resulting in two main types: modern production breeds and ancient heritage breeds. Production breeds are valued for their adaptability and high yields of milk, meat, or wool, supporting contemporary agricultural systems. Heritage breeds, on the other hand, provide unique genetic traits and resilience, having adapted for centuries in challenging conditions [10]. This dichotomy explains both the economic importance of specialized breeds and the need to conserve the genetic diversity of heritage breeds [11].

In Central Italy, productive breeds such as the Comisana are commonly used for their higher productivity in terms of milk and meat [12]. However, there is a growing effort to revive heritage breeds to preserve biodiversity and ensure sustainable farming practices, fostering a balance between modern agricultural efficiency and the conservation of traditional livestock breeds [13].

The Comisana breed originated from a crossbreeding of Mediterranean breeds with Sicilian sheep indigenous to the Comiso area, Sicily [12], in the late 19th and early 20th centuries. Characterized by its large stature, the Comisana sheep primarily excel in milk production. Currently, 69 Comisana farms are recorded in Italy, predominantly located in central and southern regions, with the highest concentration in Sicily. The total population amounts to 2870 registered animals [12].

The Sopravissana breed originated from a breeding program initiated around Visso, in the Marche region, in the 1700s, involving the crossbreeding of Vissana ewes with Merinos di Rambouillet rams [14]. The breed is indigenous to central Italy, as the name “upper Visso” (city of Visso, in the Sibillini Mountains National Park area) suggests. Sopravissana sheep are recognized for their multipurpose vocation within the local zootechnical context, including high-quality meat and wool and a lower milk yield [15]. There are 53 Sopravissana farms in Italy, all located in central regions, primarily in Marche and Lazio, with a total population of 5850 animals. At least one-third of these animals have been selectively bred exclusively to produce high-quality fine wool, while the remaining are also used for meat and milk production [14]. In Mediterranean regions, sustainable livestock management relies on three interconnected pillars: economic, environmental, and social sustainability [16,17]. In this context, breed choice may be a key management strategy, as breeds differ in productivity, adaptation to local environmental conditions, and resilience to environmental stressors.

During foraging, sheep display a clear preference for plant species of higher nutritional quality while actively avoiding poorly digestible and unpalatable species and a strong preference toward grasses over herbs [18,19], as well as a heterogeneous spatial use of the pasture and repeated use of preferred areas [20]. Spatial use and movement patterns can be influenced by several factors, including pasture management and stocking rate [21], climatic conditions [22], spatial experience of the animals [23], and forage availability [24]. These factors contribute to shaping how animals move within and use the available grazing environment. However, movement represents only one component of animal performance under semi-extensive farming conditions. Alongside movement, body condition and productive traits can provide complementary information for evaluating animals under semi-extensive farming conditions. Body Condition Score (BCS) is a widely used indicator of the nutritional and physiological status of sheep and is closely related to production characteristics [25], while milk yield represents a major productive trait in dairy sheep [26]. Accordingly, in the present study, animal movement, body condition, and milk production were considered together to compare Comisana and Sopravissana sheep under semi-extensive farming conditions.

Considering the distinct evolutionary backgrounds of the productive Comisana breed and the autochthonous Sopravissana, it is important to evaluate how breed choice can influence the sustainability of pastoral systems. We hypothesized that the distinct backgrounds and productive characteristics of the two breeds could be associated with differences in body condition, milk production, and movement under local semi-extensive farming conditions. Despite the crucial role of breed-specific traits for economic, environmental, and social sustainability in Mediterranean pastoralism, comparative studies on breed performance and adaptability remain limited.

Therefore, the study focused on three main traits: movement during free-grazing periods, body condition, and milk production, comparing Comisana and Sopravissana sheep under semi-extensive farming conditions.

## 2. Materials and Methods

### 2.1. Study Area

**Figure 1:**
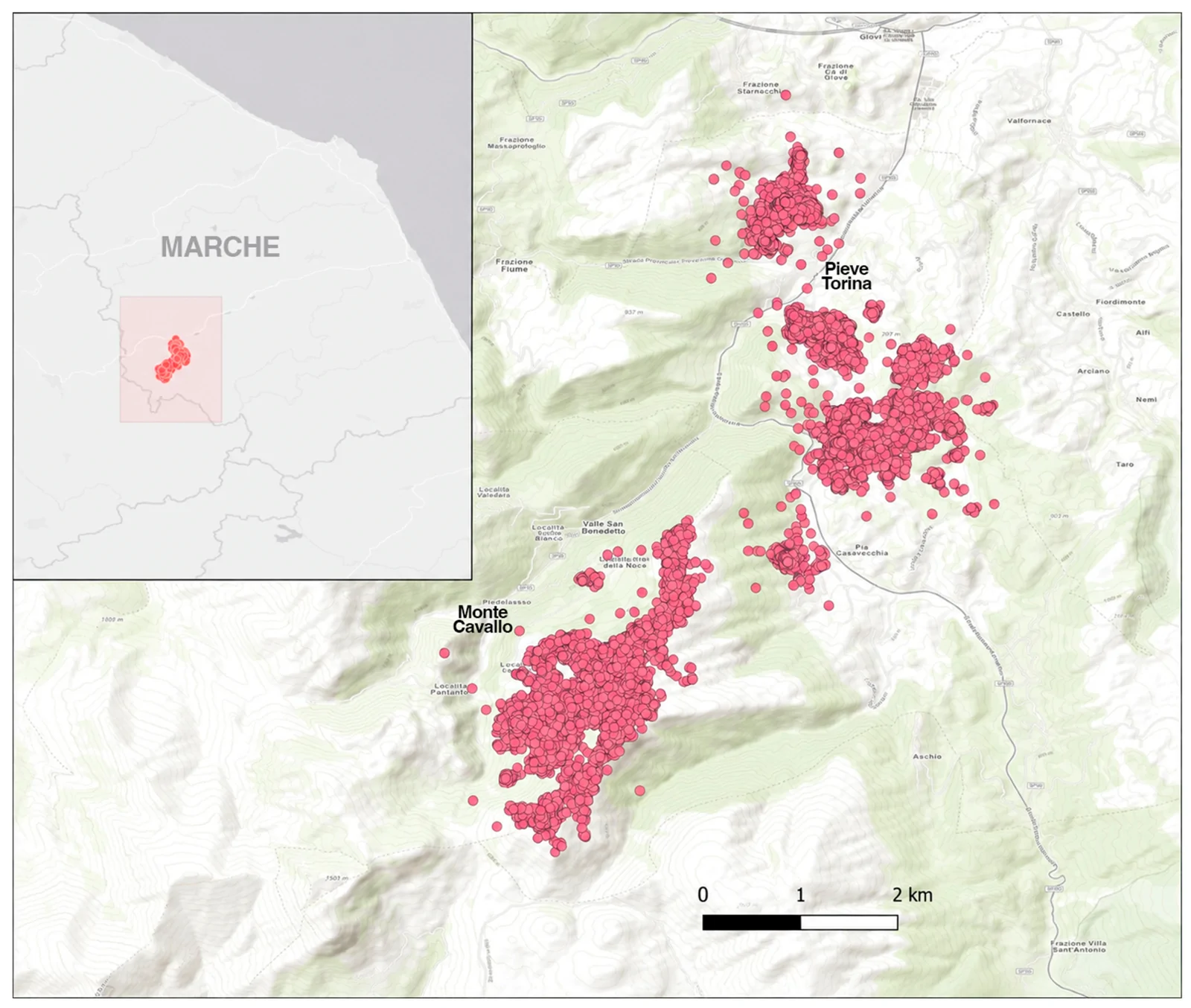
Areas of the pasture covered by the sheep. Each point represents the GPS location of a single animal at a specific moment.

The study was conducted in Central Italy, in the Sibillini Mountain National Park area (42.959–43.067° N, 12.995–13.075° E, Figure 1). The Monti Sibillini National Park is located between two Italian regions, Marche and Umbria. The extent of the area is approximately 697 km^2^, which includes numerous mountains above 2000 m, the highest of which is Monte Vettore at 2476 m, where the average altitude of the area is 1173 m a.s.l. The animals are farmed in Pieve Torina, Macerata Province, Italy. The area consists of hilly grasslands, ranging in elevation from a minimum of 500 m a.s.l. to a maximum of 750 m a.s.l.

The study area belongs to the temperate sub-Mediterranean bioclimatic unit and is characterized by winter cold stress and moderate summer drought [27,28]. The area extends across the lower supra-temperate and upper meso-temperate belts, with mean annual temperatures ranging from 9 to 13 °C and annual precipitation from approximately 850 to 1300 mm. The growing season ranges from 150 to 210 days per year [29]. The landscape is mainly characterized by secondary grasslands, with small wooded patches and croplands. Grassland communities belong mainly to the *Erysimo-Jurineetalia bocconei* and *Arrhenatheretalia elatioris* orders [29,30]. Vegetation composition varies according to slope exposure: north-facing slopes support more mesophilous communities dominated by hemicryptophytes and forbs with a prolonged flowering season, whereas south-facing slopes are characterized by more xerophilous and open grasslands, rich in annual and chamaephytic species.

### 2.2. Experimental Design

The study included complementary sampling designs according to the variables investigated, as detailed in the following sections. Animals were monitored from June to the end of October 2023.

All the sheep were homogeneous adult females, 3 years of age, and had previously completed two births (one lamb per birth). Lambing occurred between 17 and 21 February. Lambs were weaned on 22 May. The lactating sheep remained in lactation until the end of August and were subsequently dried off in September before the mating period. All animals were managed by the farm “Delizie dei Fratelli Angeli” on the same pasture. All animals were subjected to semi-extensive pastoral management techniques inside the barn during winter and on extensive pasture from June to October, according to the suitability of the season. Before the start and at the end of the outdoor season, all sheep underwent a routine health check, according to the farm’s standard management practices, which included fecal exams for gastrointestinal parasites and an external parasite inspection. No clinical parasite infestation was detected, and no one required targeted antiparasitic therapy during the trial period. During the extensive period, the animals were farmed with rotational grazing practices. Sheep were kept overnight inside smaller resting paddocks to avoid the risk of wolf predation; they were then led by the shepherd every morning to the foraging areas and brought back every afternoon. No fences were used during free-grazing times, and the shepherd led the animals to different locations inside the pasture area following the season progression. This practice ensured the most efficient pasture exploitation.

All the experiments were carried out in accordance with relevant guidelines and regulations. The protocols were in accordance with Legislative Decree No. 146, implementing Directive 98/58/EC of 20 July 1998 concerning the protection of animals kept for farming purposes.

#### 2.2.1. Animals’ Movement

During the study period, 40 female farmed sheep were equipped with GPS-dataloggers (Model: Series 4000, Manufacturer: Max-Planck-Institute Radolfzell) weighing 69 g each. The collars were powered by a Li-Ion battery (17500) and provided 12 GPS fixes per day (every 2 h) with an accuracy of ±2.5 m. Data transmission was facilitated through the Sigfox network, and all collected data were stored and managed on the Movebank platform. Additionally, the collars were equipped with a 3-axis acceleration sensor to monitor animal activity.

Animals were divided into 4 groups:

10 lactating Sopravissana sheep;

10 dry Sopravissana sheep;

10 lactating Comisana sheep;

10 dry Comisana sheep.

Data were imported into R and then projected and filtered to exclude the period in which the animals were indoors. Breed and physiological state information were added for each animal on the Movebank platform before exporting data into R. Points too far from the study area were marked as outliers. Duplicates were then removed from the dataset. The data from collar 4828 (Comisana sheep in lactation) were excluded from subsequent analyses due to functional issues with the collar (the collar did not record any data). We calculated the distance moved, angle turned, and speed for all of the animals to conduct behavioral segmentation using the move2 R package (version 0.2.7) [31] functions mt_distance(), mt_speed(), and mt_turnangle(), and movement metrics were derived from GPS data.

#### 2.2.2. Behavioral Segmentation

Behavioral segmentation was done following the methodology described by Moscatelli et al., segmenting movement data using the Expectation–Maximization Binary Clustering (EMbC) algorithm [32].

Considering the 2 h sampling interval and the flocks’ structured routine under shepherd guidance, the HL category was interpreted as directed movement (“traveling”), since it was unlikely that the animals made abrupt directional reversals. GPS fixes were recorded every 2 h for every animal, but sampling was not synchronized, providing broader group-level temporal coverage. This allowed us to identify periods when traveling events were frequently recorded across individuals.

EMbC categories were not used to directly classify grazing or resting behavior. Instead, these states were defined based on the temporal distribution of traveling events and the known pastoral routine: “resting” corresponded to periods of inactivity before and after displacements (night enclosure and non-grazing hours), while “free grazing time” was assigned to the interval between the two main traveling phases (07:00–16:00), when flocks consistently occupied grazing areas and were not following human lead. Consequently, shepherd-guided movements to and from the grazing areas were excluded from the movement analysis.

#### 2.2.3. Animals’ Body Condition

The animals’ body condition was evaluated through the Body Condition Score (BCS) evaluation technique. Body Condition Score (BCS) provides an assessment of body reserves through the evaluation of muscle and fat deposition, is widely used as an indicator of nutritional and welfare status in sheep, and is strongly related to both pasture and ruminal features [33,34,35]. BCS monitoring was conducted on the same 40 ewes included in the GPS movement monitoring. BCS was evaluated every week (15 June, 21 June, 29 June, 7 July, 14 July, 22 July, 31 July, 7 August, 16 August, 23 August, 31 August, 12 September, and 22 September 2023) by 3 experienced observers. Each observer independently assigned a score to each of the BCS parameters for every animal. Each animal’s BCS was assessed using a numerical scale ranging from 0 to 5, following the anatomical landmarks and reference criteria described by Scocco et al. [35]. The evaluation was based on palpation and visual inspection of key body regions—primarily the lumbar area, spinous and transverse processes, and the degree of muscling and fat cover—allowing a standardized and reproducible classification of body condition. The final BCS was calculated as the mean of the scores assigned by the different observers across the four evaluated parameters. Inter-observer reliability among the three experienced observers was assessed using a two-way absolute-agreement, average-measures Intraclass Correlation Coefficient [ICC(A,3)], indicating excellent agreement among observers (ICC = 0.92). The final monitoring session (22 September 2023) was excluded from the statistical analysis because ewes classified as lactating during the monitoring period had completed lactation by that time. This exclusion ensured a consistent definition of physiological state throughout the analyzed period.

#### 2.2.4. Milk Production

Milk production monitoring was conducted on 30 lactating Sopravissana and 30 lactating Comisana ewes. The 20 ewes added to the 10 animals already included in the BCS and movement monitoring were selected to maintain comparable groups in terms of age, number of previous pregnancies, and number of lambs born. The same animals were monitored throughout the entire observation period, maintaining a constant group composition across all recording sessions. Milk production data were collected every week during the lactation period (June, July, and August) by the farmer during the evening milking session, for a total of 12 recording sessions. Milk production monitoring was conducted on the same dates as the BCS assessments. Sheep were milked with an automated milking system (Milkline NG SpA, Podenzano, PC, Italy), which did not allow individual milk-yield measurements. Therefore, total evening milk yield was recorded separately for each breed group at each milking session. Farmers were provided with a datasheet to record key information, including breed, date, milking time, evening milk yield expressed in liters (L), and the number of animals milked.

### 2.3. Statistical Analysis

#### 2.3.1. Animals’ Movement

Segmented free-grazing tracks were analyzed to evaluate the influence of several variables on GPS-derived displacement between consecutive locations. Data were examined using generalized additive mixed models (GAMMs) to account for temporal autocorrelation, with the gamm function of the mgcv package in R (version 1.9-1) [36]. Log-transformed GPS-derived displacement was set as a dependent variable in the model, while breed and physiological state were included as independent variables. Individual ID was included as a random factor to account for individual variability. Time variables, specifically hour and day, were included as smooth terms to reduce the temporal autocorrelation in the data. The interaction between breed and physiological state was included in the full model as an independent variable but then excluded during variable selection considering the Akaike Information Criterion (AIC) value. Significance was set at *p* < 0.05. Autocorrelation of the residuals was visually examined to check for temporal autocorrelation using the acf function of the R stats package (version 3.6.2). No autocorrelation was detected. To verify model assumptions, residuals were inspected by plotting them against fitted values, allowing the detection of potential patterns or deviations from homoscedasticity using the gam.check function of the mgcv package in R (version 1.9-1) [36]. The residuals showed no clear trends or heteroscedasticity. The distribution of residuals was visually inspected using histograms and Q-Q plots.

#### 2.3.2. Animals’ Body Condition

Body Condition Score (BCS) data were analyzed using a linear mixed-effects model fitted by restricted maximum likelihood (REML) using the nlme package (version 3.1.169) [37]. BCS was squared prior to analysis to improve residual normality and homogeneity of variance. Breed (Comisana and Sopravissana) and physiological state (lactating and dry) were included as fixed effects, while animal ID was included as a random intercept to account for repeated measurements within individuals.

Time was treated as a continuous variable and expressed as the number of days elapsed since the first day of monitoring (day 0). To account for potential non-linear temporal changes in BCS, time was modelled using second-order orthogonal polynomial terms, including linear and quadratic components. Interactions between breed and both the linear and quadratic components of time were included, together with the interaction between breed and physiological state.

Given the longitudinal nature of the data, residual temporal autocorrelation was evaluated by comparing a model without a residual correlation structure with a model incorporating a continuous first-order autoregressive [CAR(1)] correlation structure within each animal. The CAR(1) structure was selected because time was expressed as the actual number of days elapsed between observations, thereby accounting for the temporal spacing between repeated measurements. Model fit was compared using the Akaike Information Criterion (AIC), Bayesian Information Criterion (BIC), and a likelihood-ratio test. The model including the CAR(1) structure showed a significantly better fit and was therefore retained as the final model.

Model assumptions and diagnostics were assessed using the “performance” package (version 0.18.1) [38]. Residual normality was evaluated using check_normality() and graphical inspection of the Q–Q plot; homoscedasticity using check_heteroscedasticity(); influential observations using check_outliers(); multicollinearity among fixed effects using check_collinearity(); and model singularity using check_singularity(). Overall model diagnostics were additionally inspected using check_model(). Statistical significance of fixed effects was assessed using Satterthwaite’s approximation for degrees of freedom. Marginal and conditional R^2^ were calculated using the rsquared() function from the piecewiseSEM package (version 2.0.2) [39] to quantify the variance explained by the fixed effects and by the complete mixed-effects model, respectively. To further assess pairwise differences between each combination of breed and physiological state, a post hoc analysis was performed using Tukey-adjusted pairwise comparisons with the emmeans R package (version 1.7.5) [40]. Estimates were transformed to the original BCS scale for presentation. Plots were created using the ggplot2 R package (version 3.5.1) [41].

#### 2.3.3. Milk Production

Milk production data were analyzed descriptively because milk yield was recorded as a single aggregated value for each breed group at each recording session. For each monitored group, the mean and standard deviation were calculated across recording sessions, together with the absolute and relative changes in milk production between the first (T0) and last (T11) recording sessions. To describe temporal changes independently of differences in initial production level, milk production at each recording session was also expressed as a percentage of the corresponding T0 value (T0 = 100%). All analyses were performed using R (version 4.6.1).

## 3. Results

### 3.1. Animals’ Movements

The Generalized Additive Mixed Model (GAMM) analysis (Table 1) revealed no significant differences in GPS-derived displacement during free-grazing periods between Sopravissana and Comisana sheep breeds (Estimate = 0.00394, *p* = 0.979). Additionally, no significant difference was observed between animals in lactation and those that were dry (Estimate = 0.01119, *p* = 0.940). Significant temporal effects of time and season were identified through the smooth terms in the model. The smooth function for the hour of the day (s(hour(time))) showed a significant effect (edf = 6.854, F = 22.67, *p* < 0.001), indicating distinct daily patterns in sheep activity. Similarly, the smooth function for day of the year (s(yday(time))) also demonstrated a significant effect (edf = 8.282, F = 60.67, *p* < 0.001), highlighting pronounced seasonal variations.

### 3.2. Animals’ Body Condition

**Figure 2:**
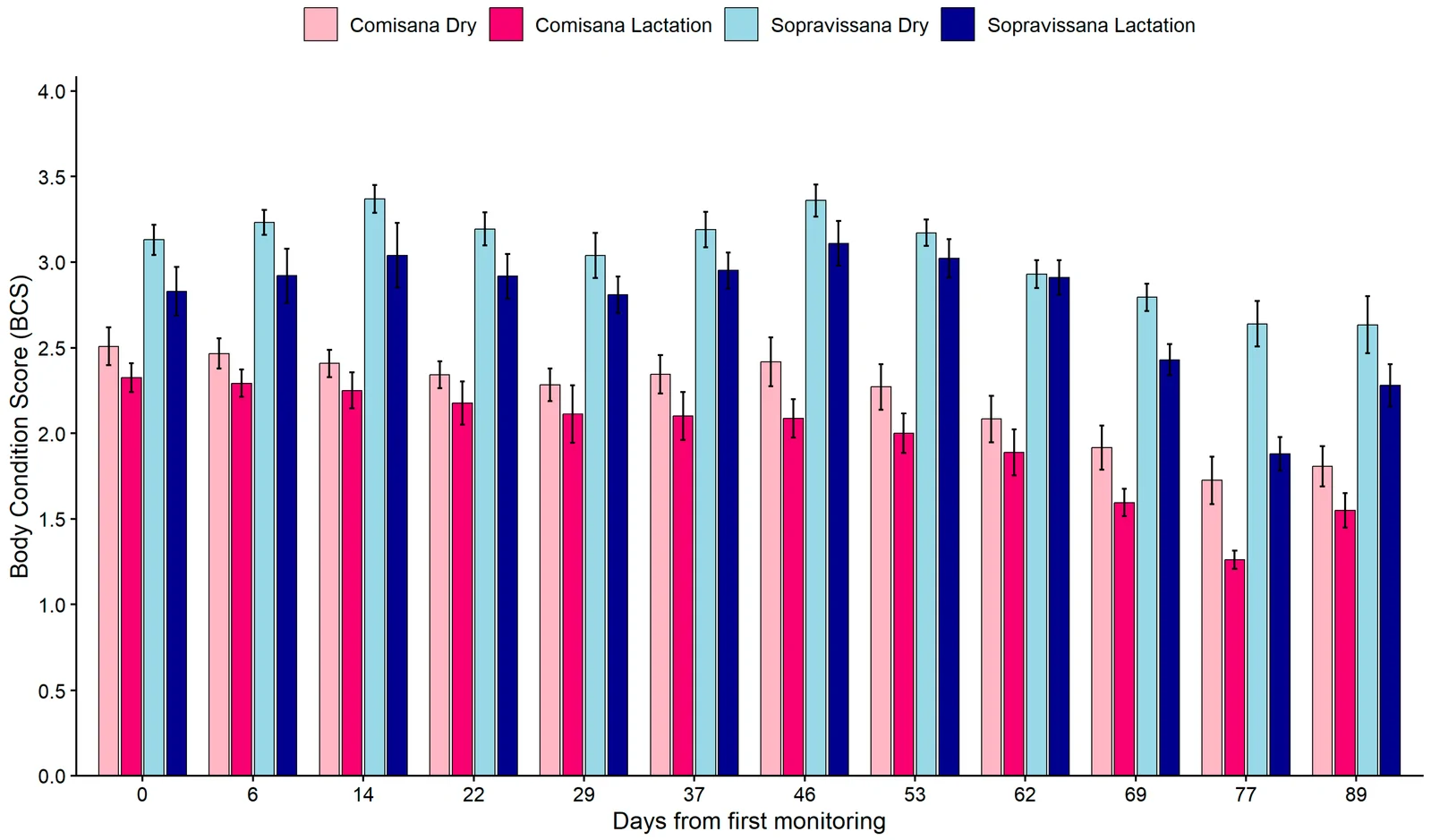
Body Condition Score (BCS) comparison between Sopravissana and Comisana sheep breeds across physiological states (Lactation/Dry).

**Figure 3:**
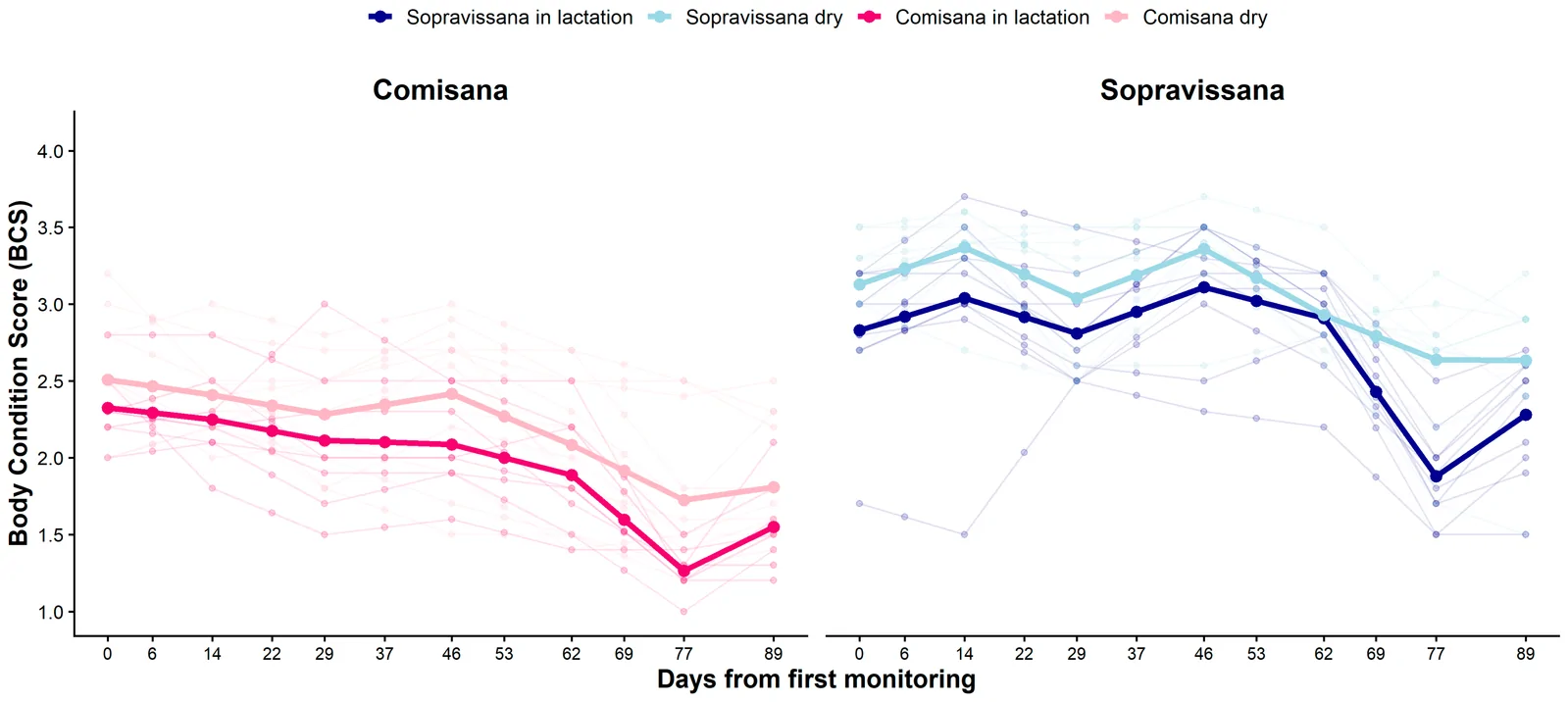
Individual temporal variation in Body Condition Score (BCS) of Sopravissana and Comisana sheep across physiological states (Lactation and Dry) during the study period. Each line represents the BCS trajectory of an individual animal, while the group mean is highlighted for each breed and physiological state.

The evaluation of Body Condition Score (BCS) data for Sopravissana and Comisana sheep across physiological states (Lactation and Dry) highlighted differences between the two breeds at different physiological states (Figure 2), which were also evident in the individual BCS trajectories over the study period (Figure 3).

As reported in Table 2, Sopravissana sheep had a mean BCS of 2.86 (SD = 0.51, Median = 3.0, IQR = 0.6, Q1 = 2.6, Q3 = 3.2), with values ranging from 1.2 to 3.7. In comparison, Comisana sheep exhibited a lower mean BCS of 2.22 (SD = 0.46, Median = 2.3, IQR = 0.4, Q1 = 2.0, Q3 = 2.4), with values ranging from 1.0 to 3.2. Lactating Sopravissana sheep showed a mean BCS of 2.80 (SD = 0.52, Median = 3.0, IQR = 0.7, Q1 = 2.5, Q3 = 3.2), while lactating Comisana sheep had a mean of 2.19 (SD = 0.44, Median = 2.3, IQR = 0.5, Q1 = 2.0, Q3 = 2.5). Dry Sopravissana sheep demonstrated a mean BCS of 2.94 (SD = 0.47, Median = 3.0, IQR = 0.6, Q1 = 2.7, Q3 = 3.3), while dry Comisana sheep had a mean BCS of 2.26 (SD = 0.48, Median = 2.3, IQR = 0.5, Q1 = 2.0, Q3 = 2.5).

The temporal variation in BCS across the study period is further detailed in Table 3. Sopravissana sheep generally maintained higher mean BCS values than Comisana sheep in both physiological states. A marked decline was observed during August, particularly in lactating animals, with the lowest mean BCS values recorded on 31 August in both Sopravissana (1.88 ± 0.10) and Comisana (1.26 ± 0.05) lactating ewes. Mean BCS subsequently increased in September.

The linear mixed-effects model showed a significant effect of breed on BCS, with higher values observed in Sopravissana than in Comisana ewes (*p* < 0.001). A significant linear temporal component was detected (*p* < 0.001), whereas the quadratic component of time was not significant (*p* = 0.383). The interaction between breed and the linear component of time was not significant (*p* = 0.919), whereas the interaction between breed and the quadratic component of time was significant (*p* < 0.001), indicating that the non-linear temporal pattern of BCS differed between the two breeds. Physiological state did not significantly affect BCS (*p* = 0.140), and no significant interaction between breed and physiological state was detected (*p* = 0.522) (Table 4).

The post hoc comparison (Table 5) highlighted several significant differences in Body Condition Score (BCS) between the various group combinations. Specifically, there was a significant difference between dry Comisana and dry Sopravissana, with dry Comisana showing a lower BCS (estimate = −0.908, t = −7.092, *p* < 0.0001). A significant difference was also observed between dry Comisana and Sopravissana in lactation, with dry Comisana again exhibiting a lower BCS (estimate = −0.637, t = −4.805, *p* = 0.0002). For the comparison between dry Sopravissana and Comisana in lactation, dry Sopravissana had a higher BCS (estimate = 1.146, t = 7.323, *p* < 0.0001). Comisana in lactation also showed a lower BCS compared to Sopravissana in lactation (estimate = −0.876, t = −5.462, *p* < 0.0001). No significant differences were observed within breeds between physiological states, either between dry Comisana and Comisana in lactation (estimate = 0.238, t = 1.485, *p* = 0.4570) or between dry Sopravissana and Sopravissana in lactation (estimate = 0.270, t = 2.454, *p* = 0.0849).

### 3.3. Milk Production

**Figure 4:**
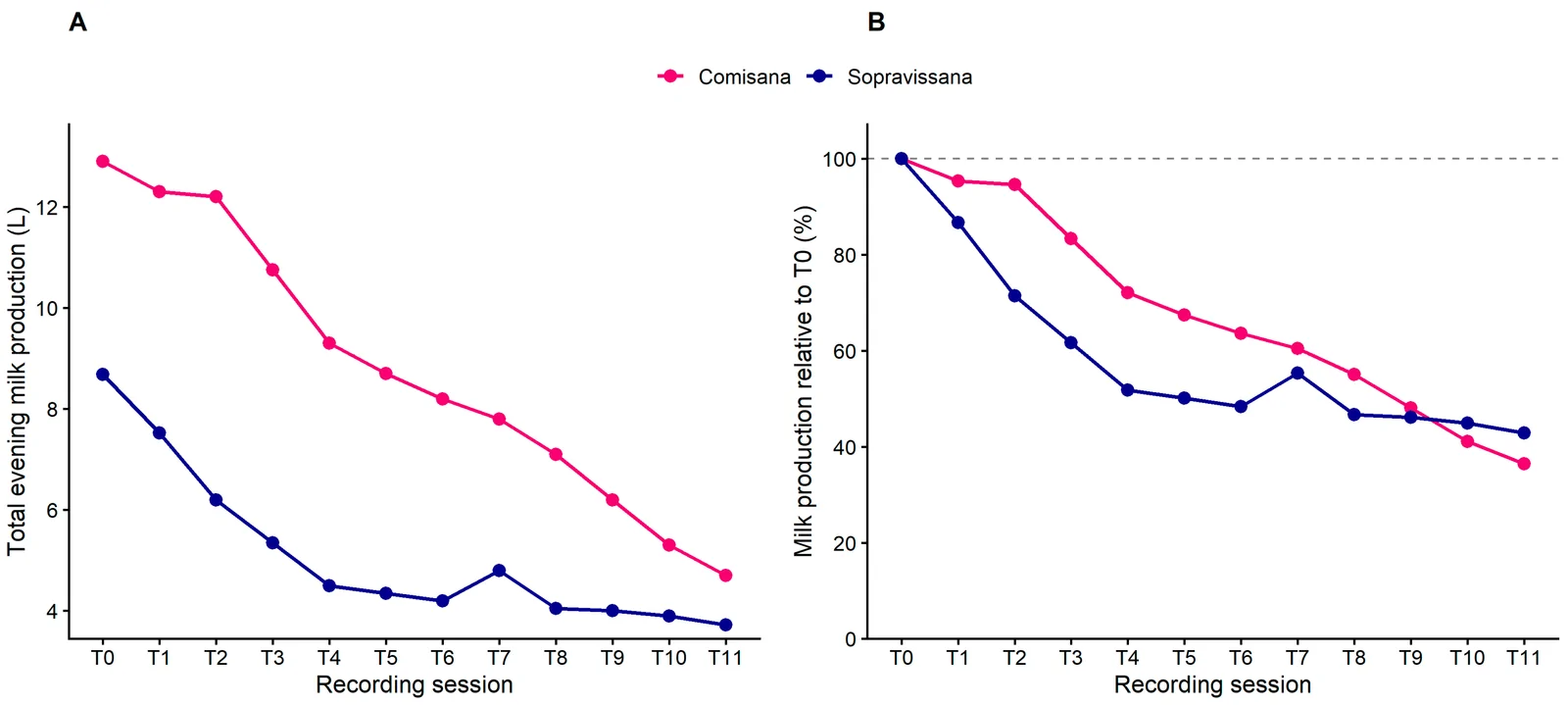
Temporal patterns of total evening milk production in the monitored Comisana and Sopravissana groups over the 12 recording sessions (T0–T11). (**A**) Total evening milk production (L) recorded for each group at each recording session. (**B**) Milk production expressed relative to the value recorded at T0 for each group (T0 = 100%). Lines connect consecutive observations, and points represent the recorded group-level values.

Across the monitoring period, the Comisana group showed higher absolute milk production than the Sopravissana group, with mean values of 8.79 ± 2.78 L/session and 5.11 ± 1.58 L/session, respectively (Table 6; Figure 4). Milk production decreased from 12.90 L at T0 to 4.70 L at T11 in the Comisana group, corresponding to a 63.6% reduction relative to the initial value, and from 8.68 L to 3.72 L in the Sopravissana group, corresponding to a 57.1% reduction. When milk production was expressed relative to T0 (T0 = 100%), different temporal patterns were observed in the two monitored groups. The Sopravissana group showed a more pronounced decline during the first part of the monitoring period, followed by a comparatively stable production level, whereas the Comisana group showed a more progressive decline throughout the observation period. At T11, milk production corresponded to 42.9% of the initial value in the Sopravissana group and 36.4% in the Comisana group.

## 4. Discussion

This study should be considered a set of preliminary observations, useful for planning more in-depth investigations aimed at selecting a higher-performing sheep breed capable of withstanding the negative effects of water stress and aridity associated with global warming.

Our findings revealed differences between Sopravissana and Comisana sheep breeds under semi-extensive farming conditions during the summer season. The two groups showed different patterns in body condition and milk production dynamics over the outdoor season, despite no significant differences being observed in movement patterns during free grazing. In the pastoral systems context, our findings highlight the importance of considering breed-specific traits when developing sustainable management strategies, as they can represent a useful tool for addressing the growing challenges associated with climate change-induced drought stress.

BCS significantly decreased over time in both groups, while the absence of a significant interaction between the variable breed and the linear component of time indicated that the linear component of the temporal trend did not differ significantly between groups. However, the significant interaction between the variable breed and quadratic time indicated that the non-linear temporal pattern differed between groups. Sopravissana ewes started from a higher BCS, and their values remained higher than those of Comisana throughout the study period. The most notable result was that Sopravissana ewes in lactation exhibited significantly higher BCS values than dry Comisana, a relevant observation considering that lactating animals usually use the majority of their body reserve for milk production [42,43]. At the last monitoring session before the mating period, the lowest mean BCS recorded in Sopravissana was 2.28, remaining above the ovine BCS threshold value of 2.0 considered suitable for successful reproduction [25,44,45]. In contrast, Comisana ewes had a mean BCS before the mating period of 1.55, below the recommended threshold. The higher body condition of the Sopravissana group provided a greater margin before reaching values considered suboptimal for health and reproduction. Overall, BCS decreased over time, although the pattern observed throughout the grazing season was not strictly linear. BCS values remained relatively stable or increased during the first part of the grazing season, followed by a marked decline in late summer and a partial recovery in September. Although pasture phenology and forage availability were not directly assessed in the present study, this pattern is consistent with the seasonal dynamics previously described for Apennine sub-Mediterranean pastoral systems, where maximum pasture productivity occurs around the flowering peak and is followed by a progressive reduction in available phytomass and forage nutritional quality toward the period of maximum summer dryness [45]. Previous studies conducted under comparable sub-Mediterranean grazing conditions have shown that seasonal and interannual variations in pasture quantity and quality can be reflected in sheep body condition, with the most pronounced reductions in BCS occurring during the late-summer mating period [35,45].

In extensive Mediterranean pastoral systems, where forage availability is influenced by seasonal fluctuations and increasing drought conditions, adequate body condition is essential in supporting reproductive success, offspring growth, and overall productivity, including wool and milk production [25,45]. Animals in optimal conditions likely require fewer interventions, such as supplementary feeding or veterinary treatment, reducing costs and improving farm efficiency [46,47].

Different temporal patterns were also observed in milk production between the two animal groups. As expected, Comisana ewes produced a higher quantity of milk than Sopravissana ewes; this result is consistent with the productive specialization of this breed [12]. Milk production decreased over time in both breed groups, with the Sopravissana group showing a less pronounced overall reduction than the Comisana group during the monitoring period. Even if the study was carried out only on one farm, it seems to indicate that the trajectories differed in shape, with the Sopravissana group showing a more pronounced decline during the first part of the monitoring period followed by comparatively stable production, whereas the Comisana group showed a more progressive decline.

No significant differences in movement during free grazing were detected between the two breeds. Similarly, Jørgensen et al., using GPS collars to compare the summer area use of two sheep breeds under alpine grazing conditions, found no significant effect of breed on the area used [48]. This similarity in movement during free grazing may be explained by the strong flocking behavior typical of sheep, as animals from both breeds tended to move collectively during grazing [49]. Considering that the two breeds grazed together as a single flock, their social cohesion may have reduced the expression of breed-specific differences in movement patterns. Moreover, the 2 h GPS sampling interval allowed us to describe broad-scale movement patterns but not fine-scale grazing behavior.

Climate projections indicate that Mediterranean mountain regions will experience a rise in temperature and an increase in drought frequency, which will directly impact water availability and consequently reduce pasture productivity and increase seasonal variability in forage availability [35,50]. Under these conditions, the better body condition observed in the Sopravissana group, in particular during lactation and at the mating period, may be relevant for maintaining animals in adequate condition under a semi-extensive farming regime, since adequate BCS is associated with reproductive and productive performance [25]. These differences highlight the importance of considering body condition alongside productive potential when evaluating breed choice in pastoral systems.

The Sopravissana breed is characterized by a triple purpose, combining wool, milk, and meat production, which represents an additional advantage in terms of income diversification and resource optimization [14]. The productive value of Sopravissana should not be assessed exclusively in terms of milk volume but also considering the potential quality and market value of its different products [51]. Economic sustainability is essential for ensuring the persistence of heritage breeds and the continuity of traditional pastoral systems [52,53]. In this view, investigating potential differences in the nutraceutical properties of dairy products among breeds, together with an economic assessment of Sopravissana cheese market value, may help to enhance farm profitability [51,53,54].

In addition, continued grazing management maintains open habitats, preserves soil health, helps control shrub invasion, and supports the biodiversity mosaics characteristic of Mediterranean ecosystems [1,3,6,7,53,55]. Preserving local breeds may also contribute to maintaining traditional farming practices and cultural heritage [13,52].

Overall, these findings suggest that welfare-related traits, such as BCS, production dynamics, and behavioral patterns, should be integrated into the assessments of pastoral system sustainability, not treated as secondary indicators.

Our findings could open perspectives for further research and practical applications. GPS locations were recorded every 2 h to preserve battery life, which may have impacted the resolution of fine-scale movement patterns and behavioral interpretations. Future studies should therefore be based on higher-frequency tracking to better capture detailed behavioral responses under extensive conditions. Milk production was recorded at the breed-group level rather than individually, preventing a biologically replicated comparison between breeds and the assessment of within-breed variability in milk yield. Future studies including individual milk-yield measurements would allow a more detailed assessment of within-breed variability, while studies conducted across different farms and years would help assess the consistency of the observed breed-related patterns under different environmental and management conditions. Future research integrating animal physiology, product quality, and farm-level economic assessments will be essential to identify management strategies capable of combining animal welfare, environmental sustainability, and economic viability in future pastoral systems.

## 5. Conclusions

Although preliminary, our findings suggest that the Sopravissana breed, characterized by its triple purpose (wool, milk, and meat), may represent a valuable resource for Apennine pastoral systems. The higher BCS observed in the Sopravissana group throughout the grazing season, including during lactation and before the mating period, together with the different milk production observed at the group level, highlights breed-specific characteristics that may be relevant under extensive summer conditions. These characteristics may become increasingly important when considering breed choice in pastoral systems facing the environmental challenges associated with climate change. However, the conservation and valorization of this breed will require innovative farm management strategies and the enhancement of its derived products. Future research should further investigate morpho-functional traits associated with farm productivity and apply product valorization approaches to Sopravissana-derived products.

## Figures and Tables

**Table 1:** Parametric coefficient estimates from the Generalized Additive Mixed Model (GAMM) assessing the effects of breed and physiological state on GPS-derived displacement during free-grazing periods. The reference categories for breed and physiological state are Sopravissana and dry period, respectively.

Term	Estimate	Std. Error	t-Value	Pr (>|t|)
(Intercept)	5.90143	0.13074	45.139	<0.001
Comisana	0.00394	0.14876	0.026	0.979
Lactation	0.01119	0.14940	0.075	0.940

**Table 2:** Body Condition Score (BCS) data for the two sheep breeds, Sopravissana and Comisana, across the two physiological states: Lactation and Dry over the outdoor season.

Group	Mean BCS	SD	Median	IQR	Q1	Q3	Range
Sopravissana	2.86	0.51	3.0	0.6	2.6	3.2	1.20–3.70
Comisana	2.22	0.46	2.3	0.4	2.0	2.4	1.00–3.20
Lactating Sopravissana	2.80	0.52	3.0	0.7	2.5	3.2	1.20–3.52
Lactating Comisana	2.19	0.44	2.3	0.5	2.0	2.5	1.00–2.80
Dry Sopravissana	2.94	0.47	3.0	0.6	2.7	3.3	1.50–3.70
Dry Comisana	2.26	0.48	2.3	0.5	2.0	2.5	1.20–3.00

SD = standard deviation, IQR = interquartile range, Q1 = quartile 1, Q3 = quartile 3.

**Table 3:** Mean Body Condition Score (BCS) of Sopravissana and Comisana sheep according to physiological state (Lactation and Dry) throughout the study period. Values are expressed as mean ± SE. Day represents the number of days elapsed since the first monitoring session (Day 0).

Day	BCS Sopravissana Lactation	BCS Sopravissana Dry	BCS Comisana Lactation	BCS Comisana Dry
0	2.83 ± 0.14	3.13 ± 0.09	2.33 ± 0.08	2.51 ± 0.11
6	2.92 ± 0.16	3.23 ± 0.07	2.29 ± 0.08	2.47 ± 0.09
14	3.04 ± 0.19	3.37 ± 0.08	2.25 ± 0.11	2.41 ± 0.08
22	2.92 ± 0.13	3.19 ± 0.10	2.18 ± 0.13	2.34 ± 0.08
29	2.81 ± 0.11	3.04 ± 0.13	2.11 ± 0.17	2.28 ± 0.10
37	2.95 ± 0.10	3.19 ± 0.10	2.10 ± 0.14	2.35 ± 0.11
46	3.11 ± 0.13	3.36 ± 0.09	2.09 ± 0.11	2.42 ± 0.14
53	3.02 ± 0.11	3.17 ± 0.08	2.00 ± 0.11	2.27 ± 0.13
62	2.91 ± 0.10	2.93 ± 0.08	1.89 ± 0.13	2.08 ± 0.14
69	2.43 ± 0.09	2.79 ± 0.08	1.60 ± 0.08	1.92 ± 0.13
77	1.88 ± 0.10	2.64 ± 0.13	1.26 ± 0.05	1.72 ± 0.14
89	2.28 ± 0.12	2.63 ± 0.17	1.55 ± 0.10	1.81 ± 0.12

**Table 4:** Results of the linear mixed-effects model (LMM) assessing the effects of breed, physiological state, linear and quadratic components of time, breed × time interactions, and breed × physiological state interaction on Body Condition Score (BCS) in Comisana and Sopravissana sheep. Model coefficients are reported on the squared BCS scale used for the analysis.

Fixed Effect	Estimate	SE	df	T-Value	*p*-Value
Intercept	5.131	0.436	435	11.765	<0.001
Breed (Sopravissana)	4.397	0.647	36	6.799	<0.001
Time—linear component	−21.158	2.851	435	−7.420	<0.001
Time—quadratic component	−1.992	2.280	435	−0.873	0.383
Physiological state (Lactation)	−1.034	0.686	36	−1.508	0.140
Breed × Time—linear component	−0.411	4.043	435	−0.102	0.919
Breed × Time—quadratic component	−11.435	3.237	435	−3.532	<0.001
Breed × Physiological state (Lactation)	−0.621	0.960	36	−0.647	0.522

**Table 5:** Post hoc pairwise comparisons of Body Condition Score (BCS) between breed and physiological-state combinations. Estimates are reported on the original BCS scale.

Contrast	Estimate	SE	df	t-Ratio	*p*-Value
Comisana Dry—Sopravissana Dry	−0.908	0.128	36	−7.092	<0.0001
Comisana Dry—Comisana Lactation	0.238	0.161	36	1.485	0.4570
Comisana Dry—Sopravissana Lactation	−0.637	0.133	36	−4.805	0.0002
Sopravissana Dry—Comisana Lactation	1.146	0.157	36	7.323	<0.0001
Sopravissana Dry—Sopravissana Lactation	0.270	0.110	36	2.454	0.0849
Comisana Lactation—Sopravissana Lactation	−0.876	0.160	36	−5.462	<0.0001

**Table 6:** Descriptive summary of total evening milk production in the monitored Comisana and Sopravissana groups over the 12 recording sessions. Mean milk production is reported as mean ± standard deviation (SD). T0 and T11 represent the first and last recording sessions, respectively. Absolute and relative changes were calculated between T0 and T11, with relative change expressed as a percentage of the initial milk production at T0.

Breed	Mean ± SD (L/session)	T0 (L)	T11 (L)	Absolute Change (L)	Relative Change (%)
Comisana	8.79 ± 2.78	12.90	4.70	−8.20	−63.6
Sopravissana	5.11 ± 1.58	8.68	3.72	−4.96	−57.1

## Data Availability

All GPS data used during the study are available on Movebank (study name “Transhumance and climate Marche 2023”, 2023–2024). BCS and production data are considered sensitive information belonging to the participating farm and therefore cannot be made publicly available.

## Abbreviations

The following abbreviations are used in this manuscript:

BCS	Body Condition Score
EMbC	Expectation–Maximization Binary Clustering
